# Microbe Mail: A microbiology and infectious diseases podcast for clinicians and students

**DOI:** 10.4102/sajid.v38i1.570

**Published:** 2023-12-21

**Authors:** Vindana Chibabhai, Gert J.K. Marais, Vinitha Alex

**Affiliations:** 1 Department of Clinical Microbiology and Infectious Diseases, Faculty of Health Sciences, University of the Witwatersrand, Johannesburg, South Africa; 2 Department of Microbiology Laboratory, Charlotte Maxeke Johannesburg Academic Hospital, National Health Laboratory Service, Johannesburg, South Africa; 3 Department of Medical Microbiology, Faculty of Health Sciences, University of Cape Town, Cape Town, South Africa; 4 Department of Medical Microbiology Laboratory, Groote Schuur Hospital, National Health Laboratory Service, Cape Town, South Africa; 5 Department of Microbiology, Ekurhuleni Region, National Health Laboratory Service, Johannesburg, South Africa

**Keywords:** medical podcast, medical education, microbiology, infectious diseases, infection prevention, control

## Abstract

Microbe Mail medical podcast is an audio-only informal educational platform, which addresses widespread topics in medical microbiology and infectious diseases (ID), with a particular focus on low- and middle-income settings. Podcasting has become a common form of informal learning in healthcare education and for continual professional development (CPD).

**Contribution:**

In this article, we discuss the development of the Microbe Mail podcast, its informal microbiology and ID education impact in the 2 years since commencement and future directions to improve uptake in Africa and low- and middle-income countries.

## Introduction

The word podcast is the result of the coalescence between the words iPod and broadcast. Despite only becoming official in 2004 as a medium for enthusiastic creators to record and disseminate information, the concept has been around since the 1990s.^1,2^

Podcasts can be considered audio versions of blogs. Audio recordings have been used in medical education since the late 1960s. Besides being used as a supplementary tool to conventional teaching in medical institutions, podcasts are also used by several leading medical journals to promote and disseminate research.^2,3^ Podcasts have varied contents, lengths and formats. They are informal yet informative platforms allowing for the rapid dissemination of information and providing expert opinions in a nutshell. In this fast-paced world, podcasts provide a medium through which information can be assimilated while listening anywhere and anytime, be it while performing chores like cleaning, cooking, exercising or even while stuck in traffic. Listeners can pick and choose topics relevant to their field of medicine and gain insight into local and global practices. While some podcasts serve to bust myths and help provide new perspectives to the persistently evolving world of medicine, others help to stay current and up-to-date on trends in the medical fraternity.

In a constantly developing world, medical podcasts serve as a novel pedagogy that can help improve medical knowledge while catering to individuals with different levels of knowledge, from medical students, nurses and pharmacists to doctors. In a study performed on medical trainees, podcast listening was reported to yield higher learning gain and was preferred to reading.^4^ By aligning to the Bloom’s taxonomy of learning, podcasts can help listeners reconstruct their way of thinking at various levels.

While there are some challenges with podcasts, such as the need for internet connectivity, the need to understand the language of the speakers, the lack of instant interaction with the speakers and, in some cases, the lack of peer review of the content, most podcasts are free or inexpensive, making them an affordable option to gain knowledge while listening to researchers and experts in their respective fields of medicine.^4^

Several healthcare disciplines such as Emergency Medicine, Internal Medicine, Neurology, Ophthalmology and Dentistry have podcasts that are produced internationally. Podcasts dedicated to Infectious Diseases (ID) and Medical Microbiology are limited both globally and in South Africa. Overall, there is a paucity of podcasts that cater to low- and middle-income countries (LMIC).^5^ With this in mind, we aimed to develop a podcast that would cover everyday issues in microbiology and ID, including diagnostic dilemmas, antibiotic advice and infection control and management conundrums that are relevant to students and busy practitioners, particularly focusing on LMIC.

## Methods

An online search of available podcasts targeting clinicians and students was undertaken to determine if a need for Microbiology and ID podcast existed in South Africa. In July 2021, an anonymous online (Google Forms) survey was conducted to determine interest, optimal format, episode length and frequency of episode release. This survey was shared with Microbiology and Infectious Diseases contacts and on multi-disciplinary clinical WhatsApp groups with requests to forward on to other clinicians and students. The survey was open for responses from 21 July 2021 to 02 August 2021.

A costing exercise was performed to determine the feasibility of implementing the podcast in the absence of a funding source.

Training on design and development of a podcast was undertaken through captivate.fm. This included determining the optimal episode length, optimal release day and time as well as frequency of content release. A podcast was set up through captivate.fm (podcast hosting platform), which disseminates podcast content widely to 18 podcast platforms. This includes Apple podcasts, Spotify, Google podcasts and Amazon Music. As part of the Captivate subscription, a podcast webpage was set up (https://microbemail.captivate.fm/). The show also required a title and logo.

Microbe Mail was designed to be an informal learning tool in the format of a relaxed, conversational style interview between host (± co-host) and an expert guest/s. Modern exclusively produced theme music adds to the informal nature of the show.

Promotion of the new podcast was undertaken through the release of a trailer describing the show and publishing this on the newly developed website and on all podcast hosting platforms (through Captivate). In order to extend the reach of Microbe Mail, social media pages were set up on Twitter (@microbemail), instagram (microbe_mail), Facebook (Microbe Mail). Episode release information was also posted on LinkedIn.

In order to ensure that the podcast produced valuable content, the design of the show and individual episodes included the quality indicators (QI) of postgraduate medical e-learning as suggested by de Leeuw and colleagues.^6^ Educational outcomes based on Kirkpatrick’s model were incorporated after the release of individual episodes.^1^

The first episode of Microbe Mail podcast was released on 14 September 2021. Fortnightly releases occurred for the first year. Thereafter, this was changed to 3-weekly releases. Bonus episodes were released for urgent public health-related issues such as the multi-country cholera outbreak in 2023.

In order for the listener to obtain further information on the topic, each episode is released with show notes which include links to articles or guidelines discussed by expert guests during the episode. The guest experts’ biographies and social media links are also included for listeners.

Continuous professional development (CPD) point allocation based on multiple choice questions (MCQ) of a small subset of episodes was trialled through the Essential Medicine (EM) Guidance platform in 2022.

## Results

### Survey findings

One hundred and eighty-five medical professionals and students responded to the survey, with 137/185 (74.1%) respondents answering yes and 46/185 (24.9%) answering ‘maybe’ to the question ‘Would you be interested in listening to a Microbiology and Infectious Diseases podcast regularly?’. The survey responses also aided in determining the optimal time to release, episode length and the themes of the episodes.

### Content and utilisation measures

As of 01 August 2023, Microbe Mail has released a total of 39 episodes covering a wide range of topics and interviewed 35 local and international expert guests. The expert guests include microbiologists, ID specialists, specialists from other disciplines with an interest in infection, laboratory technologists/technicians and infection prevention and control practitioners.

**FIGURE 1:**
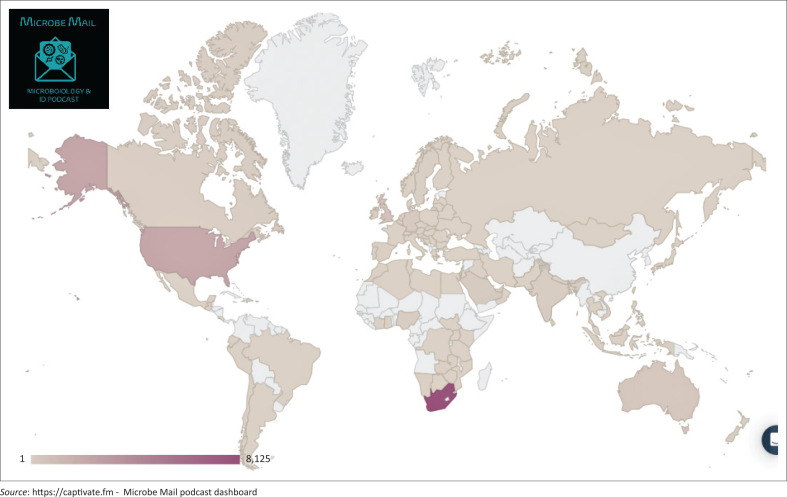
Global distribution of Microbe Mail episode downloads from 14 September 2021 to 31 July 2023.

As of 01 August 2023, Microbe Mail had a total of 8153 unique listeners and 14 817 downloads. Figure 1 shows the global distribution of episode downloads from inception until 31 July 2023. The majority of downloads were from South Africa (8125/14 817; 54.8%), followed by the United States of America (2540/14 817; 17.1%) and the United Kingdom (630/14 817; 4.2%). Microbe Mail has been listened to in 102 countries, 21 of which are on the African continent. However, only 438/14817 (2.9%) of downloads were from other African countries.

### Dissemination and visibility

As of 01 August 2023, the podcast social media pages combined have 1754 followers. To improve reach to new audiences, hashtags are used with new episode releases. The most commonly used hashtags in Microbe Mail social media posts include ‘#microbiology’, ‘#infectiousdiseases’, ‘#meded’ (i.e. medical education), ‘#infection control’, ‘#antimicrobial resistance’ and ‘#antimicrobialresistance’. Depending on the theme of the episode, applicable hashtags are used.

Microbe Mail has also engaged in collaborative episodes with other podcasts, including the ‘ID_IOTS podcast’, based in the United Kingdom ‘Let’s Talk Micro’ based in the US and the ‘Dr Coffee’ podcast in South Africa.

### Educational outcomes

To aid learning of the podcasts’ listeners, a trial of CPD point allocation based on a post-listening assessment (using MCQ) was trialled for three episodes through the EM Guidance platform (www.emguidance.com). From May 2022 to July 2023, a total of 1481 CPD points were earned by listeners through the EM Guidance platform. Table 1 outlines our assessment of the podcast’s educational impact against Kirkpatrick’s model.

**TABLE 1:** Displays our assessment of the podcast’s educational impact against the four criteria of Kirkpatrick’s model.

Criteria	Measurement and findings	Unresolved/future improvement
Reaction	Feedback on each episode Show rating on podcast players	Individual episode rating not available on podcast players or on the podcast website
Learning	Further questions and comments about specific episodes received by listeners via e-mail correspondence or social media. Trial of MCQ evaluation through EM Guidance (CPD points)	A clear, defined scoring process is required to gauge the learning of each participant. Post listening evaluations for all content
Behaviour	Listener feedback on behavioural change	Institutional performance measures are required (e.g. does patient management improve after listening to an episode of Microbe Mail)
Results	Not currently assessed	

CPD, continual professional development; MCQ, multiple choice questions.

### Time and costs

Planning of episodes, guest invitations, recording and episode scheduling takes place between the three team members (manuscript authors). Recordings are done on the online communication platform, Zoom (https://zoom.us). Editing of episode files was initially undertaken by the first author but was subsequently outsourced to a professional podcast editor. Total time required for each episode varies between 3 and 5 h.

The podcast has been self-funded by the article’s first author. Monthly costs include subscription to the podcast hosting platform (https://captivate.fm), e-mail marketing platform based on number of monthly subscribers (https://mailchimp.com) and audio file editing costs. The total monthly cost is approximately R1000.00.

## Discussion

This is the first podcast to our knowledge which tackles Microbiology and Infectious Diseases from the perspective of a low- and middle-income setting. It is also the first of its nature to be produced and hosted from South Africa. The show has grown steadily both locally and internationally in the 2-year period since inception. However, limited listenership on the African continent outside of South Africa has been noted.

This limited engagement in African countries other than South Africa may be because of various potential causes. Rodman and colleagues found that podcast penetration and listener engagement in LMICs were greater if presented in the most commonly spoken language and locally relevant topics, such as tuberculosis, are presented. Microbe Mail is currently exclusively presented in English and while many topics presented are relevant to the African continent, experts from other African countries have not featured.^5^ Medical practices also vary based on factors such as local epidemiology, guidelines and anti-microbial availability potentially resulting in limited utility of the generally South African-focused information presented.^7^

The availability of presented content on alternative platforms, such as YouTube, may also affect content engagement based on local preference for digital education consumption and Microbe Mail is currently presented as an audio-only podcast.^5^ The utility of Microbe Mail as an educational tool may thus be supported by a wider ecosystem of digital media such as infographics and a greater online presence with additional coverage of topics discussed on podcast episodes. Local preferences for additional media formats can be established through greater social media engagement. To support evidence-based practice, peer-review of episodes is further desirable with transparency and ease of reference and improved listener accessibility supported by real-time availability of episode transcripts. However, continuation of CPD point allocation per episode, expansion of the Microbe Mail media ecosystem and academic support such as peer-review will ultimately require more resources.

The assessment of the educational outcomes as presented in our results is rudimentary. However, there are currently no quantifiable measures of educational outcomes relating to digital educational content and appraisal thereof is thus quite challenging. Continued CPD point measurement based on a post-listening MCQ assessment is likely the most feasible calculable outcome.

The sustainable funding of a medical podcast can be challenging, particularly as expansion leads to greater administrative and content generation-related activities. In general, podcast revenue, beyond self-funding, can come from a number of sources including advertising, direct listener support, institutional support, subscriptions that give access to premium content, paid event hosting and crowd funding.^8^ Microbe Mail is aimed at healthcare providers in LMICs and listener funded models are not compatible with the podcast’s intended purpose of increasing access to medical education. Thus, institutional partnerships and crowd funding may be explored as the most ethically sound source of funding as advertising inevitably carries a risk of bias, particularly if medical products are services are advertised.^9,10^

Despite steady growth over a 2-year period, strategies for driving further growth of Microbe Mail in Africa and other LMIC should thus include engagement with stakeholders outside of South Africa, either as guests or co-hosts, with a focus on relevant issues and disease epidemiology. Content should also be cognisant of local practices and anti-microbial availability to ensure healthcare workers can apply acquired knowledge to their daily practice. If sufficient resources are available, release of content in additional languages can also be considered, particularly as artificial intelligence-based translation tools become more widely accessible.
